# A Method to Measure and Model Acoustic Emissions of Multicopters

**DOI:** 10.3390/ijerph20010096

**Published:** 2022-12-21

**Authors:** Jean Marc Wunderli, Jonas Meister, Oliver Boolakee, Kurt Heutschi

**Affiliations:** Empa, Swiss Federal Laboratories for Materials Science and Technology, Uberlandstrasse 129, 8600 Dubendorf, Switzerland; jonas.meister@empa.ch (J.M.); oboolakee@ethz.ch (O.B.); kurt.heutschi@empa.ch (K.H.)

**Keywords:** UAV, multicopter, noise, modeling, measuring

## Abstract

There is a growing interest for commercial applications of Unmanned Aerial Vehicles, but important foundations for an assessment, among others about noise, are missing. This contribution specifically focuses on a method to measure and model the sound radiation of multicopters. The emission prediction is hereby based on measurements using a multiple regression approach. An important finding is that the directivity pattern is widely independent of the rotational speed of the rotors and of the flight procedure. Consequently, the directivity pattern can be determined for a stationary hover flight, which considerably simplifies the measurement procedure. In addition to a rotational speed-dependent sound emission model for hover flight, a multicopter-specific correction term is required to account for forward flight. The validity of this approach is demonstrated based on the field measurements of three different multicopter models.

## 1. Introduction

Unmanned Aerial Vehicles (UAVs) or unmanned aircraft systems (UASs), commonly referred to as “drones or “multicopters”, are a relatively new transportation noise source in the environment. While multicopters are already widely used for leisure activities, commercial applications are still in their infancy. However, a wide range of applications is expected to emerge in the coming years, ranging from the transport of medical equipment and other goods to public safety or the collection of infrastructure data [1]. Many of these applications will take place in densely populated areas and might raise concerns in the population. As shown recently in [2,3], the primary cause of complaints are safety-, privacy-, and noise-related issues.

A recent review showed that information on the acoustical characteristics of multicopters is scarce and that common methods for measuring and modeling acoustic emissions of multicopters are lacking [4,5]. In addition, a detailed understanding of detrimental health effects due to multicopter noise is missing, namely exposure annoyance relations and influencing factors, such as visibility [4]. For licensing authorities, this represents a major challenge as the necessary basis for issuing operating permits is lacking. The ANSI Standardization Roadmap of 2020 [6] identified that no specific standards for multicopter noise are available to date, and high priority was given to that topic. In 2020, an ISO working group (ISO/TC 20/SC 16/JWG 7) “General requirement of noise measurement of lightweight and small multirotor unmanned aircraft systems (UAS)” was established, which issued a working draft for a standard method for noise measurements of UASs in August 2021 [7]. This draft standard describes measurement layouts for outdoor measurements and measurements in anechoic chambers and anechoic wind tunnels. However, it does not provide a specific method for data analysis and subsequent source modeling. The goal of the current contribution is to close this gap by introducing a multiple regression approach to derive source models based on laboratory and/or field measurement data. Specifically, we demonstrated how several assumptions on the source properties of multicopters can help to substantially simplify the measurement procedure. The validity and the limitations of these assumptions are discussed based on an application example. The aim of this work is to promote a simple and widely applicable method for deriving the acoustic properties of multicopters. These emission models will serve as a starting point for noise mapping and the development of assessment principles for commercial multicopter operations.

The paper is structured as follows: In Section 2, the emission model and the approach on how to gather source data is described. Section 3 briefly introduces the simulation environment used to simulate multicopter flights. In Section 4, an application case is presented, where laboratory measurement data of three different multicopters are used for the parametrization of an emission model. The corresponding source models are then used for a simulation and comparison with field measurement data of horizontal overflights. The pros and cons of the proposed concept are discussed in Section 5, and, finally, conclusions are drawn in Section 6.

## 2. Modeling the Acoustic Emissions of Multicopters Based on Measurements

### 2.1. General Flight Characteristics of Multicopters and Their Sound Radiation

In the following, some general observations about multicopters and their sound radiation properties are listed, which are a precondition for the modeling approach described in Section 2.2:

- Multicopter operations can be divided into different flight phases or procedures: forward, hover, climb, and descent flights. As multicopters typically accelerate very quickly, they can almost instantaneously switch from one operational condition to another. Consequently, at least for noise mapping purposes, the transition phases can be neglected, and only flight phases with constant operational conditions are considered. However, as these unsteady transition phases might lead to increased annoyance reactions [4], this simplification is not justified for auralization purposes [8,9].
- The sound radiated from multicopters can either be assigned to rotational noise or vortex noise [10]. While vortex noise is typically broadband, rotational noise consists of pure tone components. The blade-passing frequency represents the fundamental frequency f of the tonal components. It depends on the rotational speed of the rotors, expressed as revolutions per minute (RPM) and the number of blades of the different rotors $n_{\mathrm{Blades}}$ , as shown below.

(1) $$f=\frac{\mathrm{RPM}}{60}n_{\mathrm{Blades}}$$

**Figure 1:**
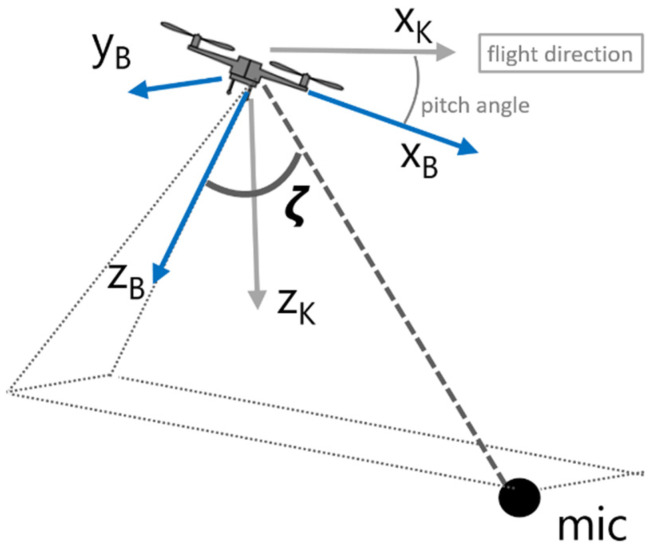
Multicopter in forward flight with the body-fixed and kinematic frames indicated by subscripts B and K, respectively. The radiation angle ζ is defined as the angle between the body-fixed *z*-axis and the line connecting the source and receiver.

- The RPM of the individual rotors of a multicopter are subject to continuous fluctuations to stabilize the multicopter and to compensate for turbulence. The amount of fluctuation is also strongly influenced by the flight procedure and is, for example, much larger during descent compared to climb [9].
- Under the assumption that the center of gravity is equidistant to all rotors, the time-averaged RPM should be the same for all rotors during hover, descent, and climb. However, to create a forward motion, the back rotors operate at a higher RPM than the ones in front. As a consequence, the multicopter is tilted in the direction of the flight, described by the pitch angle (see Figure 1).
- The sound power of multicopters depends on the RPM of the rotors. With increasing RPM, not only does the overall sound power grow, but also a spectral shift toward higher frequencies occurs. Given the strong tonal components that shift proportional to RPM, sound power can also decrease with increasing RPM in certain frequency bands.
- In addition, sound power is also influenced by the flight procedure, with typically higher sound emissions during forward flight compared to hover. This finding, based on flight experiments, is likely caused by a turbulent interaction of the airflow with the multicopter body or interference of the downwash of one rotor with other rotors [11].
- Multicopters feature a characteristic directivity pattern with higher sound radiation downward as compared to the side [9,12]. It has also been observed that the directivity pattern is more pronounced for higher frequencies. However, it appears that the directivity pattern is largely independent of the flight procedure as well as the RPM of the multicopter rotors.

### 2.2. Source Modeling Approach

Based on the above-listed observations and on previous experience of the authors with the analysis of noise measurement data of multicopters [4,8,9], we postulate the following modeling assumptions for the acoustic emission of multicopters:

- For receiver points not too close, the multicopter emission can be assumed to be radiated by a single point in the center of all rotors.
- The primary influencing parameter to describe the sound power of a multicopter is the RPM of its rotors. The sound power dependency on RPM is modeled with a second-order polynomial as given by Equation (2)
(2) $$\mathrm{Lw}\left(f,\zeta ,\mathrm{RPM},\mathrm{proc}\right)={\mathrm{Lw}}_{\mathrm{Ref}}\left(f\right)+a\left(f\right)\;\zeta^{2}+b\left(f\right)\;\left|\zeta \right|+c\left(f\right)\;\mathrm{RPM}+\;d\left(f\right)\;{\mathrm{RPM}}^{2}+C(\mathrm{proc}),$$
  where f is the center frequency for one-third octave bands from 50 Hz to 10 kHz; ζ is the radiation angle, and C(proc) is a procedural correction that accounts for sound power differences between hover and forward flights besides the RPM effect. This correction is independent of frequency, radiation angle, and flight speed (see Section 4.3).
- As outlined in Section 2.1, the multicopter sound emission consists of broadband noise and tonal components. Whereas the former is well suited for emission modeling in one-third octave bands, the latter contribution poses some challenges. Considering Equation (1), it becomes apparent that the fundamental frequency—and all higher harmonics—of the tonal emission shift proportionally with the RPM in the frequency band. For emission noise modeling in one-third octave bands, this leads to the problem that tonal components switch octave bands under large enough RPM variations. In order to remove this complicated interaction and achieve high model accuracy, this frequency shift of the pure tonal components is compensated for by normalizing the frequency of the emission spectrum by some reference ${\mathrm{RPM}}_{\mathrm{ref}}$ .
- This reference ${\mathrm{RPM}}_{\mathrm{ref}}$ can be chosen arbitrarily but should correspond to a typical operational RPM, preferably close to a mid-frequency of a one-third octave band. Sound power spectra for RPM values of interest are derived by shifting the reference spectrum proportional to the ratio between target and reference RPM. In a one-third octave band description, this transformation generally maps an original band to a target band that does not correspond to the standard filter series [13]. In order to split the power of the target band to the two standard one-third octave bands involved, it is assumed that the sound power is equally distributed within one band and can be allocated according to the fraction of the frequency range they share.
- For the directivity pattern, rotational symmetry is assumed around the body-fixed *z*-axis, leaving only the radiation angle ζ as introduced in Figure 1.
- The directivity pattern is assumed to be independent of the flight procedure and also independent of the RPM.

The model approach in Equation (2) implies that all rotors exhibit the same RPM. In situations with a significantly different RPM for each rotor, their contribution to the overall sound emission has to be modeled individually. For example, in the case of a quadcopter in forward flight, an RPM estimate for the back and front rotors is needed, which typically can be gathered from log files. Consequently, the sound power according to Equation (2) has to be divided by the share of rotors with the corresponding RPM (−3 dB in the case of two out of four rotors), and the two contributions have to be added energetically.

### 2.3. Measurement Concept

The source modeling approach as described in Section 2.2 holds the key for major simplifications of the measurement concept. Deriving full source models including directivity and RPM dependency based on fly-by measurements in the field is very laborious, time consuming, and challenging. In order to achieve a reasonable signal-to-noise ratio for the acoustic measurement, the multicopters have to pass by the microphones at a rather short distance. This in turn leads to a high sensitivity toward position uncertainties of the multicopter. In addition, the radiation angle ζ changes very rapidly during the passing of the drone in horizontal flight, so that only short averaging times can be applied to the acoustic signals. As a result, a trade-off between a sufficient time dynamic resolution and suppression of random fluctuations needs to be found. Other major sources of uncertainty are the challenging back calculations (inversion of the propagation effects), meteorological influences, and interfering background noise.

Our approach is based on a two-step procedure. First, the directivity pattern and the RPM dependency were derived based on quasi-stationary measurements. The microphone layout was comparable to the draft standard [7]. Second, simplified fly-by measurements were used to derive the procedural correction C(proc) as introduced in Equation (2).

**Figure 2:**
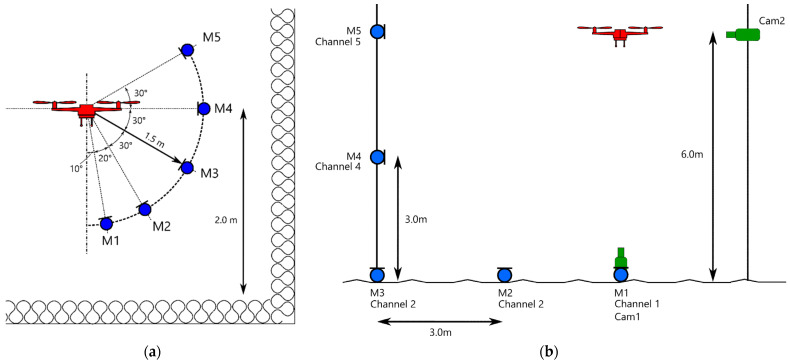
Possible measurement setups for deriving directivity and RPM dependence in the laboratory (**a**) or outside (**b**). Microphone positions are shown as blue dots. (**b**) additionally proposes two cameras in green that are used to accurately identify the position of the source.

For smaller multicopters, it is recommended to perform step one under controlled laboratory conditions and low background noise. Ideally, the multicopter is operated at a fixed position in hover mode, and the microphones are placed at different radiation angles, as shown in Figure 2a. In order to avoid a disruption of the measurement by flow recirculation, the multicopter should not be operated too close to the ground [14]. Measurements are performed at different RPM settings by varying the payload. Alternatively, the multicopter can be mounted on a tripod, which simplifies the RPM variation and control over the position. Thereby, it must be ensured and controlled that all the rotors are running at identical RPM.

The measurements should be performed in an anechoic room to avoid unwanted reflections. In addition, it is advisable to use high-performance wind screens for microphones exposed to the downwash (in particular, microphone M1 in Figure 2a). The distance of the microphones from the center of the multicopter must be chosen to be sufficiently large to justify the assumption of a point source. For larger multicopters, this might not be feasible, and outdoor source measurements should be considered.

Figure 2b shows a possible measurement layout. To achieve a good coverage of the radiation angles even with few microphones, the multicopter can be operated in hover at different heights. An optical observation of the multicopter with two cameras (horizontally and vertically) is one way of guaranteeing high positioning accuracy. Again, a variation of RPM can be achieved by different payloads.

The second step, the derivation of a procedural correction for forward flight, can be performed with a rather simple measurement setup. Essentially, only a single microphone is required that records a fly-by at a representative travelling speed. Of course, averaging over several microphones and/or flights will reduce measurement uncertainty. The microphone(s) should be placed sufficiently close to the flight path to guarantee a good signal-to-noise ratio, but far enough away to reduce measurement errors caused by position uncertainties of the source. Field measurements showed that logged GPS position data feature an accuracy in the range of few meters in the horizontal plane, but substantially larger uncertainties in the vertical direction. A position determination solely based on GPS is therefore not accurate enough, and additional methods are needed. In our specific case, a mix of optical and acoustical triangulation was used to reduce the position uncertainty.

The procedural correction C(proc) in Equation (2) was derived by comparing the measured sound exposure spectra of fly-bys with the simulation model results based on laboratory measurement data (see Section 4.3). Because the sound exposure spectrum is a time-integrated quantity, the already mentioned challenges regarding position accuracy and temporal resolution are substantially reduced.

## 3. Simulation Procedure in sonAIR

With the aim of reproducing level-time curves of individual multicopter flights and displaying noise contours caused by multicopter flights at a later stage, the sonAIR aircraft noise simulation tool [15] was extended for the application of multicopters. sonAIR follows a time-step procedure and calculates the momentary sound pressure level spectrum on the ground for specific source points along the flight path. On this basis, a wide range of acoustic metrics can be calculated, such as sound exposure or maximum sound pressure level for different time weightings. The propagation model used in sonAIR considers geometrical spreading, air absorption, Doppler frequency shift, and reflections from the ground based on an analytical solution for spherical waves, which was extended for finite segment length and variable ground properties. As meteorological effects, on the one hand, the local influence of temperature, relative humidity, and air pressure on air absorption and, on the other hand, the effect of vertical sound speed gradients on barrier effects and on the evolution of acoustical shadow zones, can be considered. Additionally, sonAIR can include buildings as obstacles and reflectors and is therefore well suited for simulating the sound exposure in urban environments, which might be of interest when modeling multicopter noise. For the integration of multicopter operations, sonAIR had to be extended in three aspects:

(1) For fixed-wing aircraft, sonAIR assumes that the aircraft is oriented in the direction of the flight track. However, as multicopters can reach large pitch angles, the body-fixed frame was chosen as a reference instead of the kinematic frame (see Figure 1).
(2) So far, flight profiles in sonAIR were defined with respect to the distance travelled. To enable the simulation of hover phases, the flight profiles can now optionally be defined with respect to elapsed flight time as well.
(3) A semiempirical flight mechanics model to estimate the RPM of rotor pairs based on the multicopter type, its payload, and travelling speed was developed by a partner institution, the Aerospace Project Development Group (ALR), and integrated in sonAIR.

The multicopter flight paths in 3D space were generated by convoluting a 2D ground track with height profiles, as well as information on horizontal and vertical flight speeds. As discussed in Section 2.1, the flight dynamic capabilities of multicopters enable rapid transitions between different flight phases. As a result, each flight segment of the procedurally generated path is modeled with constant speed. Entire flights are composed of several segments with uniform flight parameters and constant acoustic emissions. On this basis, level time histories for specific receiver locations as well as noise maps for entire areas can be calculated. 

## 4. Application Example

As an application example, laboratory as well as field measurements are presented for three multicopters, which were all provided and operated by the company Meteomatics (see Table 1).

### 4.1. Source Modeling Based on Measurements in the Laboratory

Source measurements were performed on 17 June 2019 in an anechoic room at the authors’ institution, using a microphone setup as shown in Figure 2a, with five microphones placed at a distance of 1.5 m from the center of the source and the multicopter being placed 2 m above ground. For microphones M2 to M5, standard windscreens were used. Microphone M1, which was exposed to the downwash of the rotors, was equipped with a high-performance windscreen (Rycote, model 086014). To compensate for the additional attenuation of the latter, a correction filter was applied in the data analysis. The three multicopters under evaluation were attached to a frame. Relative to the maximum RPM of each model, the following power settings were tested: 20, 25, 40, 50, 60, 75, 80, and 100%. The RPM of a single rotor was additionally controlled using an optical sensor. However, an additional acoustic analysis of the pure tone components revealed that the RPM of the individual rotors were not uniform in all cases, with a variability of up to 15%, so that an average value over all rotors was used as a representative RPM instead. For the acoustic analysis of the pure tone components, we applied an in-house method that involved a ridge-tracking algorithm applied to the power spectral density of the multicopter audio signal.

The calibrated sound recordings of each setting were performed with a MOTU 896 mk3 audio interface, and the sound pressure levels in one-third octave bands, averaged over 30 s, were evaluated. For a given emitter–receiver distance of r= 1.5 m, the measured sound pressure level Lp was converted into a sound power level Lw by the following relation:

(3) $$\mathrm{Lw}=\mathrm{Lp}+10\;\log_{10}\left(\frac{4\pi r^{2}}{A_{0}}\right)=\mathrm{Lp}+14.51\;\mathrm{dB},$$

with the reference area defined as $A_{0}=1m^{2}$ .

**Figure 3:**
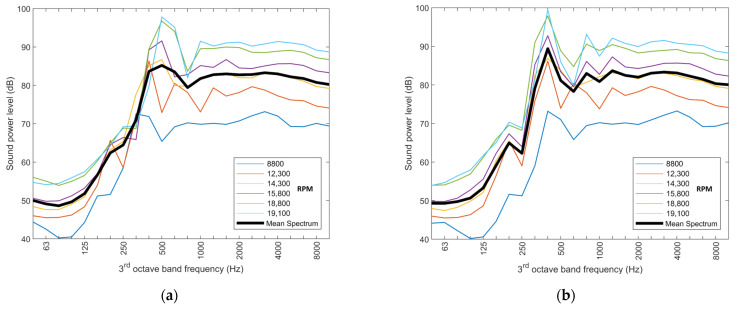
Sound power spectra of the SSE multicopter, derived from measurements at microphone M3 (radiation angle of 60°) for different RPM, (**a**) before and (**b**) after the pitch shift to the reference RPM .

Reference emission spectra were derived based on RPM values of 5400, 3800, and 12,100 for the Classic, XL, and SSE multicopters, respectively. As an example, Figure 3 shows the emission spectra of the SSE multicopter, measured for a radiation angle of 60°, before and after accounting for the pitch shift. As can be seen, the normalization of the frequency by some reference ${\mathrm{RPM}}_{\mathrm{ref}}$ leads to similar spectra for different RPM values that are only different in their amplitude. This normalization allows to assume the simple RPM dependence as in the model approach of Equation (2) without having to consider some interaction with the frequency.

**Figure 4:**
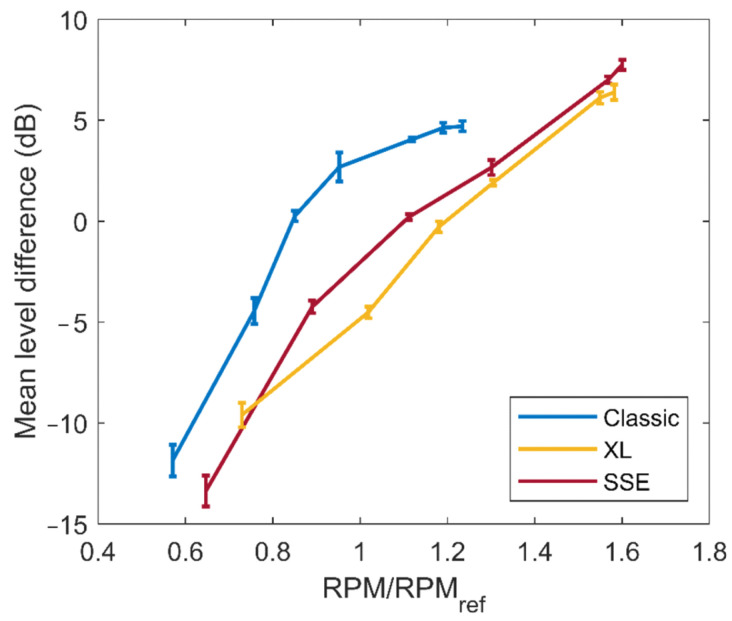
Relation between the sound power and the normalized RPM, as introduced in the source model, for the three multicopters under evaluation. The error bars (standard deviation) show the variation for all considered radiation angles.

Figure 4 shows the resulting RPM dependency for the three multicopters under evaluation. The function for the RPM dependence was thereby determined by searching for the minimum deviation of the spectrum at the ${\mathrm{RPM}}_{\mathrm{ref}}$ compared to the real RPM spectrum, equally weighted over all third-octave bands from 50 Hz to 10 kHz. The RPM dependence was derived as an average over all measured radiation angles. As can be seen from the error bars in Figure 4, the relation is thereby widely independent from the radiation angle. Consequently, it can be confirmed that the directivity pattern can be assumed constant, regardless of the RPM setting.

**Figure 5:**
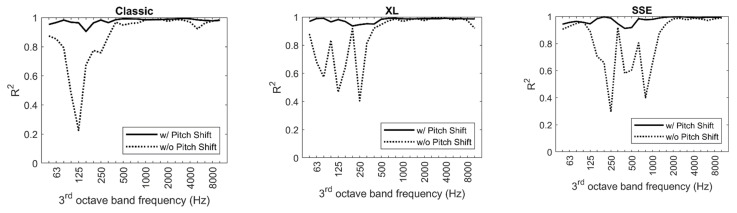
Coefficient of determination $R^{2}$ of source models for the three multicopters under evaluation, determined with and without pitch shift.

The significant improvement of the goodness-of-the-fit of the regression model by normalizing to a reference ${\mathrm{RPM}}_{\mathrm{ref}}$ is shown in Figure 5. Without the pitch shift, rather low coefficients of determination are achieved for the frequencies with strong tonal components. In contrast, accounting for this pitch shift yields very good results with $R^{2}>0.9$ for the entire frequency range and all models.

**Figure 6:**
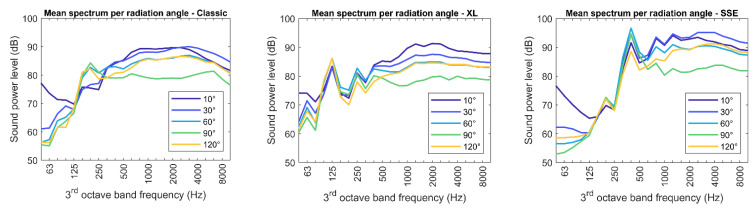
Sound power spectra in dependence of the radiation angle ζ , for the three multicopters under evaluation.

Figure 6 shows the individual directivity pattern of the three multicopters under evaluation. They were derived as an arithmetic average over all measured spectra, after applying the pitch shift transformation. As can be seen, the radiation is higher at lower emission angles, especially for frequencies above the blade-passing frequency (see Equation (1)). The lowest emission levels are typically found at 90°, i.e., in the horizontal plane. Comparing the sound power levels for radiation angles of 60° and 120° indicates that the radiation pattern is symmetrical with respect to the x–y plane of the body-fixed reference frame.

### 4.2. Horizontal Flight Experiments

Field measurements were performed on 13 November 2019 over plane, grass-covered ground using the microphone layout as depicted in Figure 2b. Microphones M1 (with windscreen Rycote, model 086014) to M3 were placed on ground plates to guarantee a sound pressure doubling from the ground reflection. The calibrated microphone signals were simultaneously recorded with a SD MixPre-10. In addition, the fly-bys were recorded with two cameras. Note that this measurement scheme went beyond the recommendations given in Section 2.3. This setup with a larger number of microphones, partially also underneath the flight path and in rather small fly-by distances, was chosen to validate several model assumptions in the field. 

During the measurements, the ambient average temperature was 5 °C, and the relative humidity was around 60%, with a partially cloudy sky. A mild west wind with an average wind speed of 2 m/s was present. As the flight path led from West–Northwest to East–Southeast, this approximately corresponded to full headwind conditions in one flight direction and tailwind in the opposite direction. When comparing the two flight directions, this wind influence only had a minor effect on RPM, but caused an average shift of the pitch angle between 5 and 10 degrees, depending on the type of multicopter.

The multicopters crossed the measurement plane spanned by the microphones (see Figure 2b) along an orthogonal flight path in both directions with a target overflight height of 6 m. The constant flight speeds (see Table 2) were reached with average deviations of ±1 km/h and maximum deviations of ±5 km/h. During the flights, GPS coordinates and performance data including RPM of all rotor engines were logged. Audio signals were recorded with all microphones for each overflight event.

During the data analysis, the position uncertainty of the flight trajectories caused by the multicopter GPS was significantly reduced by an image analysis of the camera recordings in combination with an acoustical triangulation. The position was thereby determined based on the autocorrelation function of the different microphone signals; i.e., the required offset of two signals to achieve the maximum autocorrelation value was taken as the signal time delay between these two microphones. The combination of multiple delay results between microphone pairs was used for triangulating the emitter position in space. The RPM of the individual rotors were taken from the flight logs and verified acoustically by identifying the pure tone components in the sound signal, under consideration of the Doppler frequency shift.

### 4.3. Comparison with Simulations and Determination of Procedural Corrections

The forward flight experiments were simulated using sonAIR, neglecting meteorological influences on sound propagation and initially without the procedural correction C(proc) . The flight path was discretized with a temporal resolution of 0.01 s and simulated for a duration of 8 s, symmetrically over the shortest distance. A digital terrain model of the Swiss Federal Office of Topography (Swisstopo) was used in a grid format with 1 m x 1 m resolution. Reflections from the ground were considered assuming grassy ground with the ground plates introduced as acoustically hard surfaces of $1m^{2}$ size.

**Figure 7:**
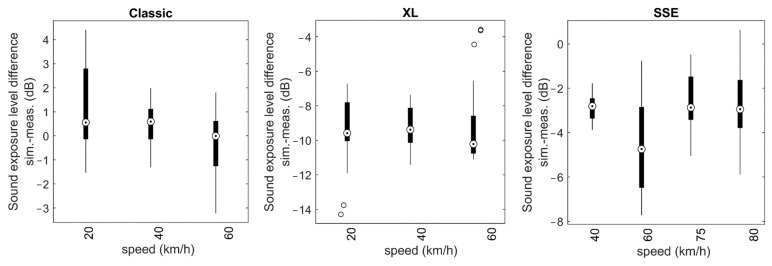
Box–whisker plots of the difference of simulated vs. measured sound exposure levels for the three multicopters under evaluation and different speeds.

Figure 7 shows the difference of simulated vs. measured sound exposure levels for the three multicopters under evaluation and different speeds as box–whisker plots. The size of the boxes varies between 1 and almost 4 dB indicating a considerable variation between events, presumably due to the influence of wind and flight path estimation uncertainties. Interestingly, when looking at the mean deviation, no clear correlation with speed is visible in the data, despite the rather large range of flown speeds. With the exception of the SSE multicopter, operated at 60 km/h, the mean deviation appears to be largely independent of the overflight speed for each multicopter. For the case of the SSE at 60 km/h, a detailed analysis showed that the three ground microphones M1 to M3 contributed most to the mentioned deviation. We therefore assume a potential underestimation of the overflight height.

In general, the absolute values of these differences vary substantially between the three multicopters, ranging from almost zero in the case of the Classic to approximately −3 dB for the SSE and almost −10 dB for the XL. Thereby, negative values indicate that the multicopter produces higher sound power levels in forward flight compared to hover with identical RPM, i.e., the condition the emission model was tuned for. These mean differences as shown in Figure 7 were used as procedural correction C(proc) for the final source models according to Equation (2).

**Figure 8:**
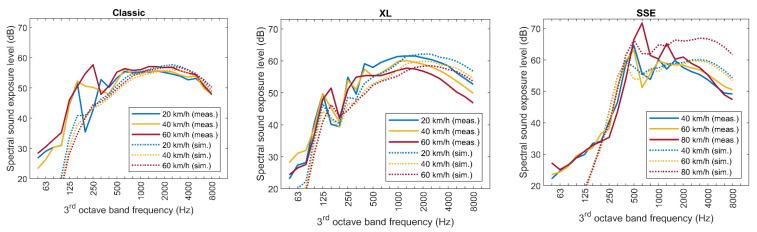
Comparison of the measured vs. simulated spectra of the sound exposure level at microphone position M4, shown for all multicopters and three different speeds.

**Figure 9:**
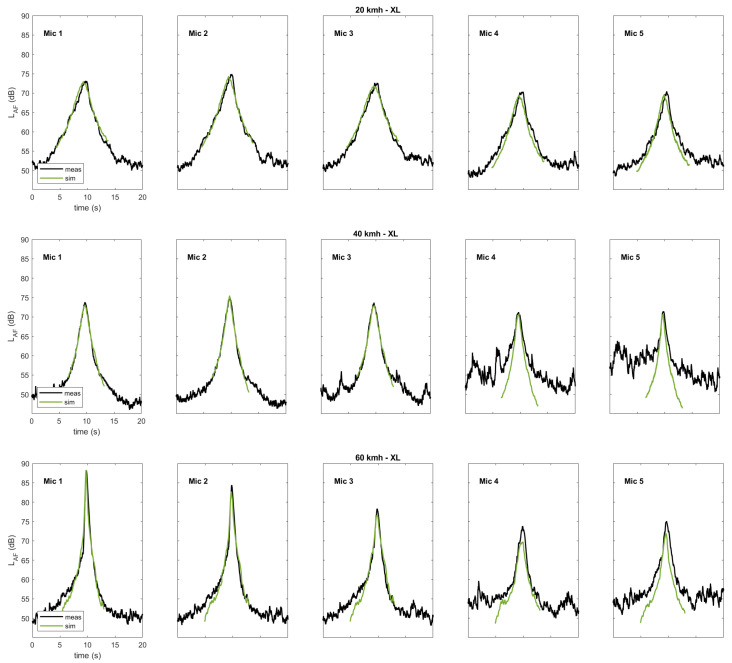
Comparison of measured vs. simulated A-weighted sound pressure level time histories for exemplary flights of the XL multicopter at three different velocities, shown for all microphones.

Figure 8 and Figure 9 show further comparisons of the simulation results with measurements, using the final model including the procedural correction. In Figure 8, the averaged sound exposure spectra are compared for three different speeds for each of the three multicopters and for microphone 4. Microphone 4 was chosen, as this geometry is most representative for typical receivers, such as a nearby house. As can be seen in the spectra shown in Figure 8, the procedural correction only partially succeeds in reducing the deviation between the model and the experiment. For the Classic, the agreement is good in the high-frequency bands beyond the tonal components of the emission spectrum. For the one-third octave band around 160 Hz, the strong increase in the sound emission level with increasing speed is only visible in the measurements, but not reproduced by the model. However, as can be seen in Figure 8, the emission strength is very low for frequencies below the fundamental tonal component. Additionally, its influence on the A-weighted sound exposure level used for noise evaluation is negligible, because of the strong attenuation of the A filter at low frequencies. The agreement for the XL is good in general, with the measurements showing stronger spectral level variations compared to the smoother spectrum of the model. For the SSE, the measured and simulated spectra agree well for 40 and 60 km/h, but particularly for 80 km/h, the simulations significantly overpredict the measured levels in the high-frequency range.

Finally, Figure 9 shows a comparison of the measured vs. simulated A-weighted sound pressure levels over time for the exemplary flights of the XL multicopter at three different velocities and for all microphones. (For completeness, the corresponding figures showing exemplary flights of the Classic and the SSE drones are given in the Appendix A) Some measurements were influenced by interfering background noise (see 40 km/h, microphones 4 and 5). However, in general, the agreement is very good for all microphone positions, not only with regard to the peak levels but also with regard to the level slopes.

The standard deviation of the model error with respect to speed and microphone position amounts to 1.5 dB for the Classic and 1.8 dB for the XL and the SSE multicopters, in terms of the A-weighted sound exposure level.

## 5. Discussion

The application example shown cannot be considered as a full model validation, as the measurement data were used to derive the procedural correction for forward flight. The measurements showed a substantial scattering, primarily due to uncertainties in the position determination or wind influences. The experiments clearly showed that the accuracy of the GPS position data provided by the log of the multicopters was not sufficient. In this project, an improvement of the position determination could be achieved by using a mix of optical and acoustical triangulation, but this aspect can and should be further improved in the future. In the field of acoustic localization, several concepts have been introduced that could serve as a starting point for that purpose [16,17,18,19]. Concerning wind influences, t as already mentioned, they were neglected in the sound propagation modeling, and it might be assumed that at least part of the substantial scattering seen in Figure 7, could be explained by this simplification. However, the rather short propagation distances in combination with the source heights of several meters above ground support the assumption that meteorological effects on sound propagation can be widely neglected. In contrast, the influence of the wind on the operational conditions of the multicopter will play an important role. This aspect should be elucidated further in future research and as suggested by Kapoor et al. [20] could be used for the optimization of flight paths.

On average, no systematic deviation between measurement and simulation was observed, indicating that the underlying model assumptions were appropriate. For example, the good reproduction of the level slopes in Figure 9 is a clear indication that the directivity pattern is correctly captured. This also confirms the assumption that the directivity pattern is largely independent of the RPM setting and flight speed. Another significant result is that the relation between the noise emission model for each one-third octave band and the RPM of the rotors heavily benefits from a normalization of the measured frequency spectrum by a reference ${\mathrm{RPM}}_{\mathrm{ref}}$ . This pitch shift is a key finding to disentangle the various influencing parameters and to end up with a simple model approach as shown in Equation (2).

**Figure 10:**
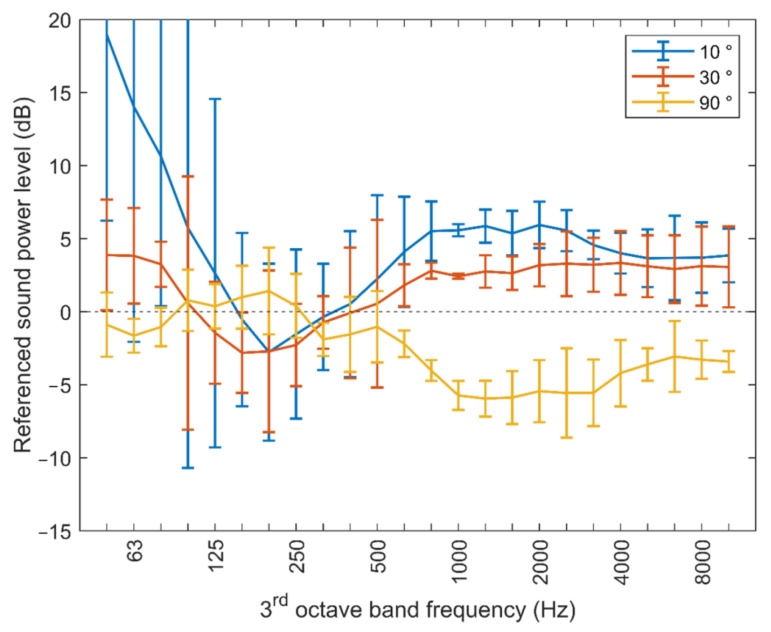
Averaged directivity pattern with the bars showing the variation among the three multicopters under evaluation. Note: The averaged directivity pattern is shown for three radiation angles with the spectrum at a radiation angle of 60° used as reference.

When comparing the directivity pattern as given in Figure 6, they are clearly very similar. In Figure 10, the averaged directivity patterns for the three multicopters including error bars that represent the whole range of the differences are shown. The strongest variation is observed for the radiation angle ζ = 10° at frequencies below the characteristic tonal components of the noise emission spectrum. However, it has to be taken into account that the overall sound emission in these low-frequency bands is fairly small. In combination with the fact that the bottom microphone is strongly affected by the rotor downwash, these high variations in the low-frequency bands can be attributed to a poor signal-to-noise ratio. For the other radiation angles and higher frequencies, the deviations are comparably small, which supports the recent proposition by Heutschi et al. assuming a uniform vertical directivity pattern independent of the specific multicopter type [8,9]. Based on the measurement data collected there, a radiation characteristic D(f,θ) [dB] depending on frequency f and direction θ=90°−ζ was found that can be described as:

$$D\left(f,\theta \right)=\frac{G\left(\theta \right)}{\pi }\;\left(\arctan(5.8\;\left(\log\left(f\right)-2.66)\right)+\frac{\pi }{2}\right)$$

with

$$G\left(\theta \right)=-0.0011\theta^{2}+0.194\left|\theta \right|-4.9$$

Although derived for a different set of drone models, D(f,θ) runs largely within the scatter bars of Figure 10 and supports the hypothesis of a generalized multicopter directionality. It should be noted that the drone models studied were predominantly quadcopters (one exception: hexacopter). We assume that the results can also be transferred to multicopters with more than four rotors. However, this proof will still have to be provided with future measurements.

Relying on such a generic radiation characteristic enables a further simplification of the measurement procedure so that in addition to the procedural correction, only the relation between the RPM setting and sound power needs to be determined for a new multicopter model.

As indicated in Section 4.3, the identification of the RPM dependence can be performed with hover experiments alone. However, in order to be able to predict the emission spectra for horizontal flight as well, a procedural correction is required. This correction turns out to be widely independent of the horizontal flight speed, but the specific value of the correction is surprisingly different for the three multicopter models investigated in this work. We can only speculate about the causes of these large differences. In the authors’ opinion, the increased noise emission observed for horizontal flight is probably a result of certain flow interactions, such as the propeller downwash with the drone body or with the neighboring rotors. This assumption is supported by the measurement results of Zawodny et al., who report level differences of up to 10 dB triggered by varying pitch angles and vertical offsets of the rotors [21]. These effects should be investigated further, as they appear to play a key role in optimizing the efficiency of multicopters and in reducing their sound emission.

## 6. Conclusions

A modeling approach in combination with a measurement concept is presented to derive acoustic source models of multicopters for environmental noise modeling purposes. The approach was successfully applied to three types of multicopters, operated at different horizontal flight speeds. The deviations in the validation are found to be random and can be attributed to the scattering in the measurements. The elegance of the approach lies in the fact that the primary influencing variables, namely the directivity pattern, the RPM dependence, and a procedural correction, are described independently of each other. This makes it possible to determine the influence of each of the parameters separately and thus simplifies both the measurement data requirements and the subsequent model generation. The application example supports the underlying assumptions and confirms that, despite the simple approach, reliable source models can be generated that are well suited for noise mapping applications. However, given the fact that multicopter noise consists of prominent tonal components, the proposed modeling approach based on one-third octave bands is not ideal for psychoacoustic studies. For such applications, a full auralization, reproducing sound pressure signals in fine temporal resolution is deemed more appropriate. To achieve results with higher accuracy in the future, on the one hand, the position measurements should be improved and, on the other hand, refined data analysis techniques for acoustic frequency tracking, position determination, and model generation (automate model approach, i.e., Greedy algorithm) should be implemented.

## Figures and Tables

**Table 1:** Multicopter type, dimensions (mm), and weight (g). (The designation of the multicopters was taken from the manufacturer).

	Classic	XL	SSE
Type	Quadcopter	Quadcopter	Hexacopter
Outer diameter [mm]	578	797	407
Rotor diameter [mm]	254	345	127
Weight with battery [g]	2850	3350	763
Blades per rotor	2	2	2

**Table 2:** Number of fly-bys per multicopter and speed in the forward flight experiment.

Multicopter	20 km/h	40 km/h	60 km/h	75 km/h	80 km/h
Classic	6	6	6	0	0
XL	6	6	6	0	0
SSE	0	4	6	4	6

**Figure A1:**
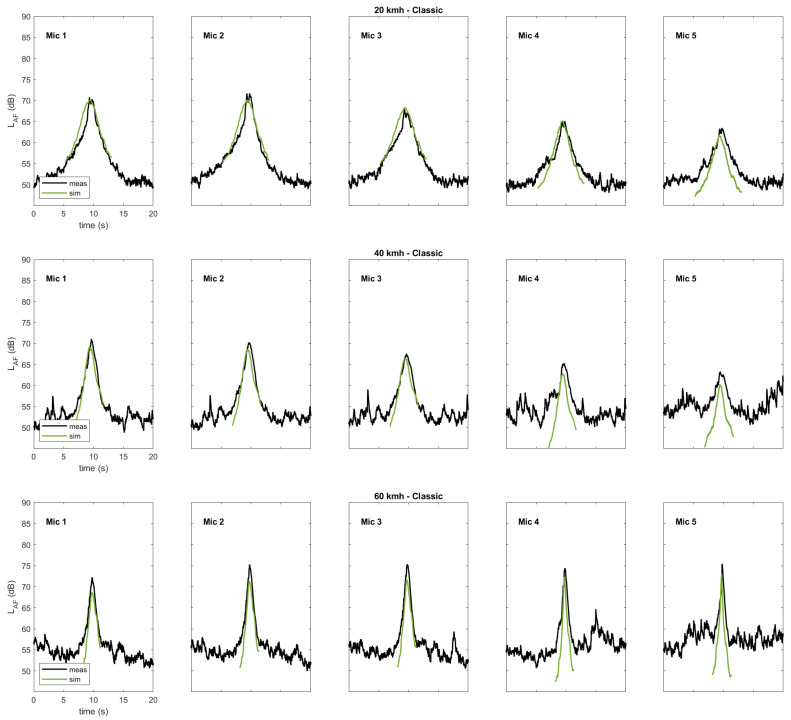
Comparison of measured vs. simulated A-weighted sound pressure level time histories for exemplary flights of the Classic multicopter at three different velocities, shown for all microphones.

**Figure A2:**
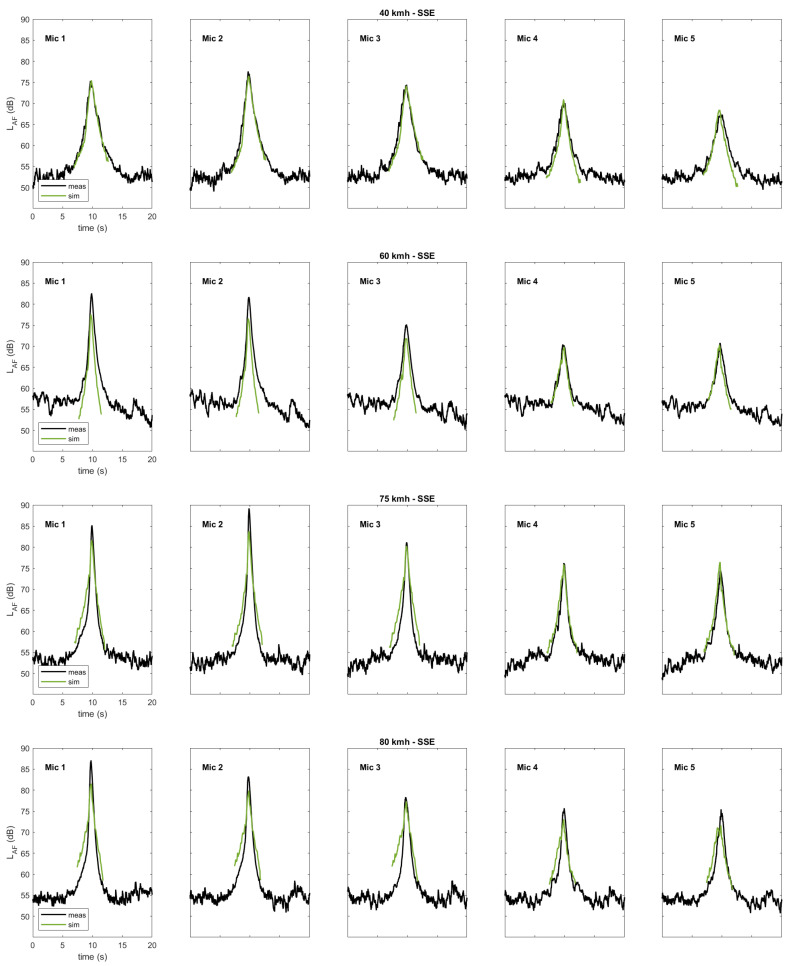
Comparison of measured vs. simulated A-weighted sound pressure level time histories for exemplary flights of the SSE multicopter at four different velocities, shown for all microphones.

## Data Availability

Not applicable.
